# Causes and outcome of acute kidney injury amongst adults patients in two hospitals of different category in Cameroon; a 5 year retrospective comparative study

**DOI:** 10.1186/s12882-022-02992-4

**Published:** 2022-11-14

**Authors:** Teuwafeu Denis Georges, Halle Marie-Patrice, Tonou Sorel Ingrid, Ronald Gobina Mbua, Fouda Menye Hermine, Ashuntantang Gloria

**Affiliations:** 1 Faculty of health Sciences, University Of Buea, P. O Box 63, Buea, Cameroon; 2 Faculty of Medicine and Pharmaceutical Sciences, University of Douala, Douala, Cameroon; 3 Faculty of Medicine and Biomedical Sciences, University of Yaounde I, Yaounde, Cameroon

**Keywords:** Acute kidney injury, Causes, Outcome, Adults, Cameroon

## Abstract

**Background:**

Acute kidney injury (AKI) is an under-recognized disorder, which is associated with a high risk for mortality, development of chronic kidney disease (CKD).

**Objective:**

We sought to describe and compare the causes and outcomes of AKI amongst adult patients in Douala general hospital (DGH) and Buea regional hospital (BRH).

**Methods:**

A hospital-based retrospective cohort analytic study was carried from February to April 2021. Convenience sampling was used. We included Patient’s files admitted from January 2016 to December 2020 aged > 18 years, with AKI diagnosed by a nephrologist and recorded values of serum creatinine (sCr) on admission and discharge. Data were analysed using SPSSv26. Chi-square, fisher, median mood’s and regression logistic test were used, values were considered significant at *p* < 0.05.

**Results:**

Of the 349 files included 217 was from DGH and 132 from BRH. Community acquired AKI were more present in BRH 87.12% (*n* = 115) than DGH 84.79% (*n* = 184) (*p* = 0.001). Stage III AKI was the most common presentation in both hospital. Pre-renal AKI was more common (*p* = 0.013) in DGH (65.44%, *n* = 142) than BRH (46.97%, *n* = 62). Sepsis and volume depletion were more prevalent in urban area with (64.51 and 30.41% vs. 46.21 and 25.75%) while severe malaria was more present in Semi-urban area (8.33% vs. 1.84%, *p* = 0.011). Complete and partial renal recovery was 64.97% (*n* = 141) in DGH and 69.69% (*n* = 92) in BRH (*p* = 0.061). More patients had dialysis in BRH 73.07% (*n* = 57) than in DGH 23.33% (*n* = 21). More patient died in DGH 33.18% (*n* = 72) died than in BRH 19.70% (*n* = 26) (*p* = 0.007). Stage III was significantly associated with non-renal recovery in both DGH (*p* = 0.036) and BRH (*p* = 0.009) while acute tubular necrosis was associated with non-renal outcome in DGH (*p* = 0.037).

**Conclusions:**

AKI was mainly due to sepsis, volume depletion and nephrotoxicity. Complete and partial recovery of kidney function were high in both settings. Patient outcome was poorer in DGH.

## Introduction

Acute kidney injury (AKI) is a heterogeneous condition that encompasses prerenal, intrinsic, and postrenal AKI, [1]. In developed countries, hospital acquired AKI is the most frequent form with an incidence of 7–18%, preponderance for elderly and up to 60% of critically ill patients [1]. While in Low and Middle Income Countries like Sub-Saharan Africa (SSA) the most frequent is community acquired affecting young adult and children [2]. The cause of AKI is often multifactorial with pre-existing comorbidities further increasing the risk [3–5], and include sepsis, volume depletion, nephrotoxic agents, major surgery, cardiogenic shock, complications with medications, hepato-renal syndrome, obstructive uropathy [5–8]. A recent meta-analysis by Kahindo et al. reported that the common causes of AKI in adults in SSA were infections (28%), nephrotoxins (18%), pregnancy related (16%), glomerular disease (8%) and hypovolemia (5%) [9].

Despite advances in medical technique, AKI remains under diagnosed especially in SSA. When diagnosed late, AKI has adverse effects for the individual in general [1]. The reported outcomes following AKI are consistently poor [4]. It is associated with increased length of hospital stay [1], increased resource utilization, and mortality. Also the risk for the development of chronic kidney disease (CKD) [10] and kidney failure (KF) is increased by 10 times and 3 times respectively [1]. In SSA, the outcome of patients with AKI is very poor with an overall mortality of 32% in adults and increases with the severity of the disease which is estimated at 50–60% amongst patients requiring kidney replacement therapy (KRT) and to 82% in those in need of dialysis who could not receive it due to late presentation, non-availability of KRT and inability to afford treatment [10].

In Cameroon, there are several studies on AKI more in tertiary health centres. In this multicentre study, we aimed at comparing the causes and the outcomes of AKI in adult in two hospital at different level of care in Cameroon representing urban and semi-urban settings.

## Patients and methods

### Study design

This was a hospital based retrospective analytic cohort study. Over 3 months, data from files of patients admitted for AKI from 1st January 2016 to 31st December 2020 were collected. Two hospitals at different levels of care in the health pyramid and serving different type of population were randomly selected: the Douala general hospital a tertiary hospital that served as reference hospital at the national level and is situated in the economic capital of the country and the Buea regional hospital a secondary hospital situated at the intermediate of the Cameroon health pyramid, that served as reference hospital for the region, and is situated in a semi-urban town. Both hospitals have nephrologists and a dialysis centre.

### Data collection

AKI was defined in this study as an increase or decrease in serum creatinine of 0.3 mg/dl or more with a baseline serum creatinine < 1.5 mg/dl or a percentage increase or decrease in the serum creatinine concentration of ≥50% within 7 days. For the baseline creatinine, whenever available, prehospitalisation Scr (community acquired AKI) or the sCr on admission (hospital acquired AKI) were used. When baseline Scr was missing, we considered absolute increase in the serum creatinine of 0.3 mg/l within 48 hours or observed 1.5 fold increase in creatinine in 7 days knowing that lack of baseline creatinine when defining AKI have shown to led to underestimation of AKI and Higher mortalit y[11]. The following data were collected from patients files; age, gender, profession, comorbidity, past history of AKI, drug history, and physical examination, and laboratory investigations that included urinalysis, blood urea, serum creatinine and serum electrolytes. Ultra-sonography of the kidneys and bladder were performed when needed for the size and the structures of the kidneys and the presence of hydronephrosis. The mechanism of AKI was determined using clinical reasoning (kidney biopsy were not performed) and the outcome studied were the hospital length of stay, kidney recovery (total or partial), need for dialysis and death.

File of patients with diagnosis of hepato-renal syndrome and severe heart failure, patients younger than 17 years, patients with known chronic kidney diseases (CKD) or features suggesting CKD, and those with incomplete clinical information were excluded.

### All methods were performed in accordance with the relevant guidelines and regulations

Data were entered into a computer then exported from Excel 2016 and analysed using the Statistical software, SPSS version 26.0 Chi-square, fisher mood’s median test were used to compare variables. Regression statistical model was used to test for significance of association between the independent and dependent variables. Values were considered significant at *p* < 0.05.

### Definition of terms

1. **AKI Staging**: The KDIGO 2012 classification was used to stage the severity [12]
2. **Community acquired AKI**: If patients was reported to have presented the in hospital with signs and symptoms of AKI or developed it less than 2 days of hospitalization.
3. **Hospital acquired AKI**: AKI developed more than 2 days of admission of patients who were admitted with normal kidney function.
4. **Sepsis**: files with clinical diagnosis of sepsis or as the presence of a proven or suspected microbial infection in the presence of at least two of the following criteria: Temperature > 38 °C or < 36 °C, pulse rate > 90 beats/minute, respiratory rate > 24 cycles/minute, white cell count > 12,000 cells/mm3 or < 4000 cells/mm3 recorded in the file.
5. **Pre-renal AKI** was defined based medical history, the presence of risk factors, urea/creatinine ratio > 20 in the absence of confounders and urine indices (specific gravity> 1020, absence of leucocytes) or recorded diagnosis when available were used.
6. **Acute tubular necrosis** was defined based on medical history, presence of risk factors, absence of leucocytes in urine, urea/creatinine ratio < 10 and the polyuria in the recovery phase. **Interstitial nephritis** was defined in the presence of leucocytes in the urine, preserved urine output, delay recovery and the absence of polyuria at the recovery phase. **Post obstructive** was diagnosed based on history of acute oliguria or anuria and the presence of hydronephrosis on ultrasound**. Acute glomeruloneph**ritis was defined for file with clinical element of nephritic syndrome
7. **Nephrotoxin induced AKI** was defined as a recorded diagnosis of AKI, that followed a history of ingestion of known nephrotoxic drug (conventional, or herbal remedies), or a recorded diagnosis of AKI due to nephrotoxic agent.
8. **Urine abnormalities** were defined as one or more of the following on dipstick: Proteinuria ≥ + 1, Leucocyturia ≥ + 1, Haematuria ≥ + 1, Nitrite ≥ + 1, Glycosuria ≥ + 1
9. **Kidney function recovery** was assessed at discharge and calculated by the ratio of sCr to baseline sCr at the time of kidney function assessment, according to the following criteria: (1) Total kidney function recovery: when creatinine returns to the sCr baseline value; (2) Partial recovery: when sCr does not return to the baseline value but stays within a margin up to 1.5 times the baseline value, no need for dialysis; (3) No recovery: sCr stays at a value above 1.5 times in relation to the baseline [13]
10. **Length of stay**: was considered from the first day of admission.
11. **Need for dialysis** was referred to patients with indications for dialysis
12. **Access to dialysis** to those with indications for dialysis and that were actually dialysed.

### Ethical consideration

Ethical clearance was obtained from the Faculty of Health Sciences, University of Buea (FHS), and its Institutional Review Board (ref. 2021/1313–02/UB/SG/IRB/FHS). The confidentiality of participants was maintained by using codes rather than names on questionnaires. Since we were conducting a retrospective study, the need for study participant consent was waived by the Ethics Unit (Institutional Review Board, Faculty of health science university of Buea).

## Results

A total of 349 patients’ files were assessed in both hospitals (217 files from DGH and 132 files from BRH); there were more male patients, 55.58% (*n* = 194), the median age (IQR) was 54 (17–93) years, one patient out of four was aged less than forty years. As shown in Table 1, the leading comorbidities were Hypertension in 29.79%(*n* = 104), Diabetes 21.20%(*n* = 74), HIV 20.34% (*n* = 71). The main nephrotoxic agent used was herbal medicine 18.62% (*n* = 65). History of AKI was present at 4.58% (*n* = 16). Majority of patients presented with fever 47.56% (*n* = 88). Abnormal urine dipstick was observed in 72% of patient with proteinuria of all degree being the main abnormality. The mean serum creatinine (SD) level was 3.68 (5.27) mg/dl (Table 2).

**Table 1:** Socio-demographic and clinical characteristics of the study population

Variable	Median (IQR)	Frequency	Percentage (%)
**Age**	54(17–93)		
< 20		5	1.43
20–39		84	24.06
40–59		134	38.39
60–79		104	29.79
>80		22	6.30
**Occupation**			
Student		78	22.35
Unemployed		96	27.51
Self-employed		86	24.64
Employed		69	19.77
Retired		20	5.73
**Symptoms on admission**			
Oliguria		88	25.21
Fever		166	47.56
diarrhoea/Vomiting		46	13.16
Body Swelling		26	7.44
Convulsion		14	4.01
Loss of consciousness		49	14.04
Hemiparesis/Hemiplegia		14	4.01
Other		13	3.72
**Comorbidity**			
HIV		71	20.34
Hypertension		104	29.79
Diabetes		74	21.20
Hepatitis b virus		15	4.29
Cancer		30	8.59
CKD		28	8.02
**History of AKI**		16	4.58
**History of gout**		14	4.01
**Toxicology**			
NSAIDS		14	4.01
ACEI		26	7.44
ARB		1	0.29
Herbal medicine		65	18.62

*CKD* chronic kidney disease, *NSAIDS* non-steroidal anti-inflammatory drugs, *ACEI* angiotensin converting enzymes inhibitors, *ARB* angiotensin receptors blockers, *HIV* human immunodeficiency virus, *IQR* Interquartile Range, *AKI* Acute Kidney Injury

**Table 2:** Para-clinical characteristics of the study population

Variable	Mean (SD)	Frequency(n)	Percentage (%)
**Urine abnormalities**			
Protein		217	62.17
Blood		118	33.81
Glucose		29	8.30
Bilirubin		11	3.15
Ketone		24	6.88
Leukocyte		98	28.08
Nitrite		66	18.91
**Serum creatinine (mg/dl) (*n*** **= 349)**	3.68 (5.27)		

**Fig. 1:**
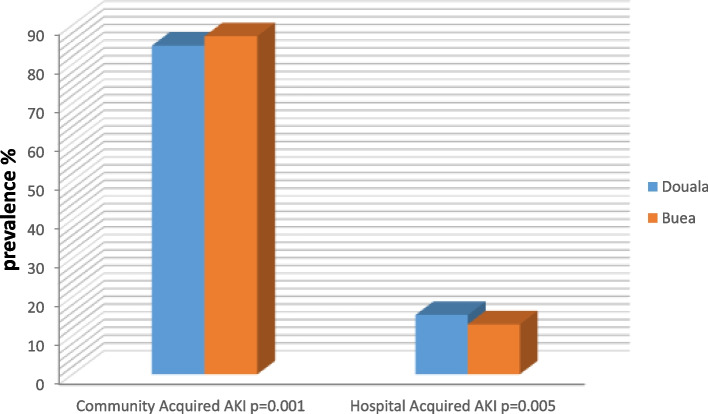
Type of acute kidney injury amongst groups of study population

Community acquired AKI was the main type of AKI reported in 85.67%. Community acquired AKI was significantly higher in BRH (87.12% vs. 79%, *p* = **0.001**). While Hospital Acquired AKI was significantly more present in DGH (15.2% VS 12.87%, *p* = **0.005**) as shown in Fig. 1. There was no difference in the distribution by severity in both hospitals and Stage III was the most frequent at presentation (58.25%) followed by stage 1 (31.35%).

The main aetiologies of AKI in both centres included: sepsis, volume depletion, nephrotoxins and malaria. Comparatively, sepsis (64.51%), volume depletion (30.41%) and pregnancy related AKI (Preeclampsia, eclampsia, HELLP syndrome) were significantly higher in DGH while Severe malaria was significantly higher (8.33% VS 1.84%, *p* = **0.011**) in BRH (Table 3).

**Table 3:** Aetiologies of acute kidney injury amongst groups of studied population

Aetiology	Total (%) ***N*** = 349	DGH (%) ***n*** = 217	BRH (%) ***n*** = 132	***p***
Sepsis	201(57.59)	140(64.51)	61(46.21)	**< 0.001**
Volume depletion	100(28.65)	66(30.41)	34(25.75)	**0.003**
Obstruction	25(7.16)	8(3.69)	17(12.88)	0.717
Nephrotoxin	30(8.60)	11(5.07)	19(14.39)	0.717
Severe malaria	15(4.29)	4(1.84)	11(8.33)	**0.011**
Malignant hypertension	16(4.58)	5(2.30)	11(8.33)	0.960
Preeclampsia/eclampsia/HELLP syndrome	12(3.44)	9(4.15)	3(2.27)	**0.019**
Multiple myeloma	1(0.29)	0(0.00)	1(0.76)	–

**Fig. 2:**
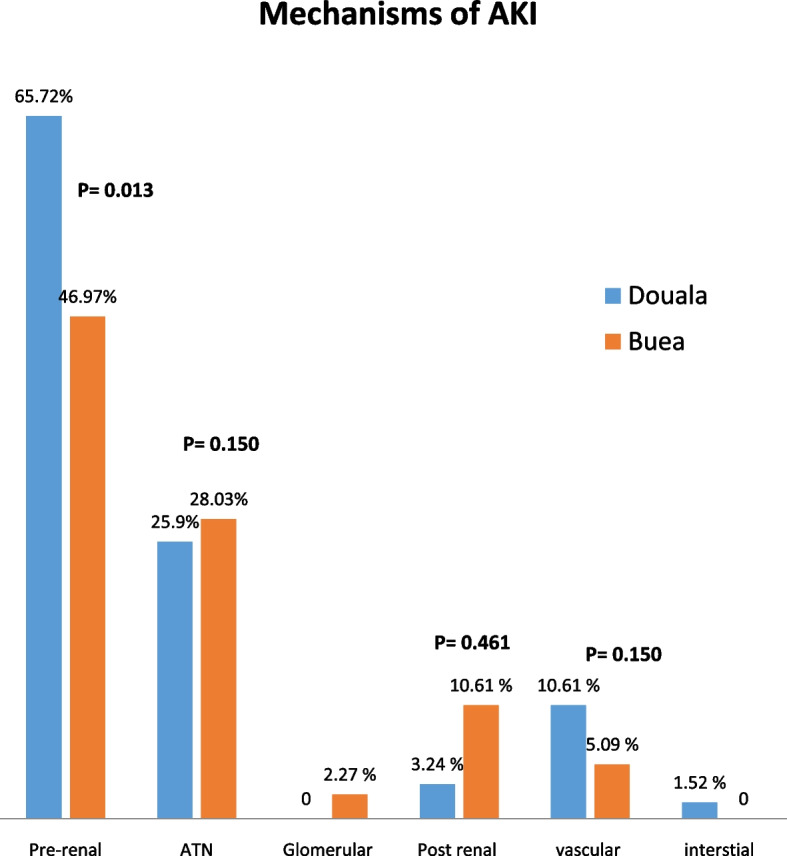
Mechanisms of acute kidney injury amongst groups of studied population

As shown in Fig. 2, Pre-renal AKI was the most common type of AKI in both centres and was present in 57.33%of patients followed by acute tubular necrosis in 27.01%. Pre-renal AKI were significantly higher in DGH (65.44% VS 46.97% *p* = **0.013**). Acute tubular necrosis accounted for 25.93% in DGH and 28.03% in BRH but the difference was not significant (*p* = **0.150**).

**Fig. 3:**
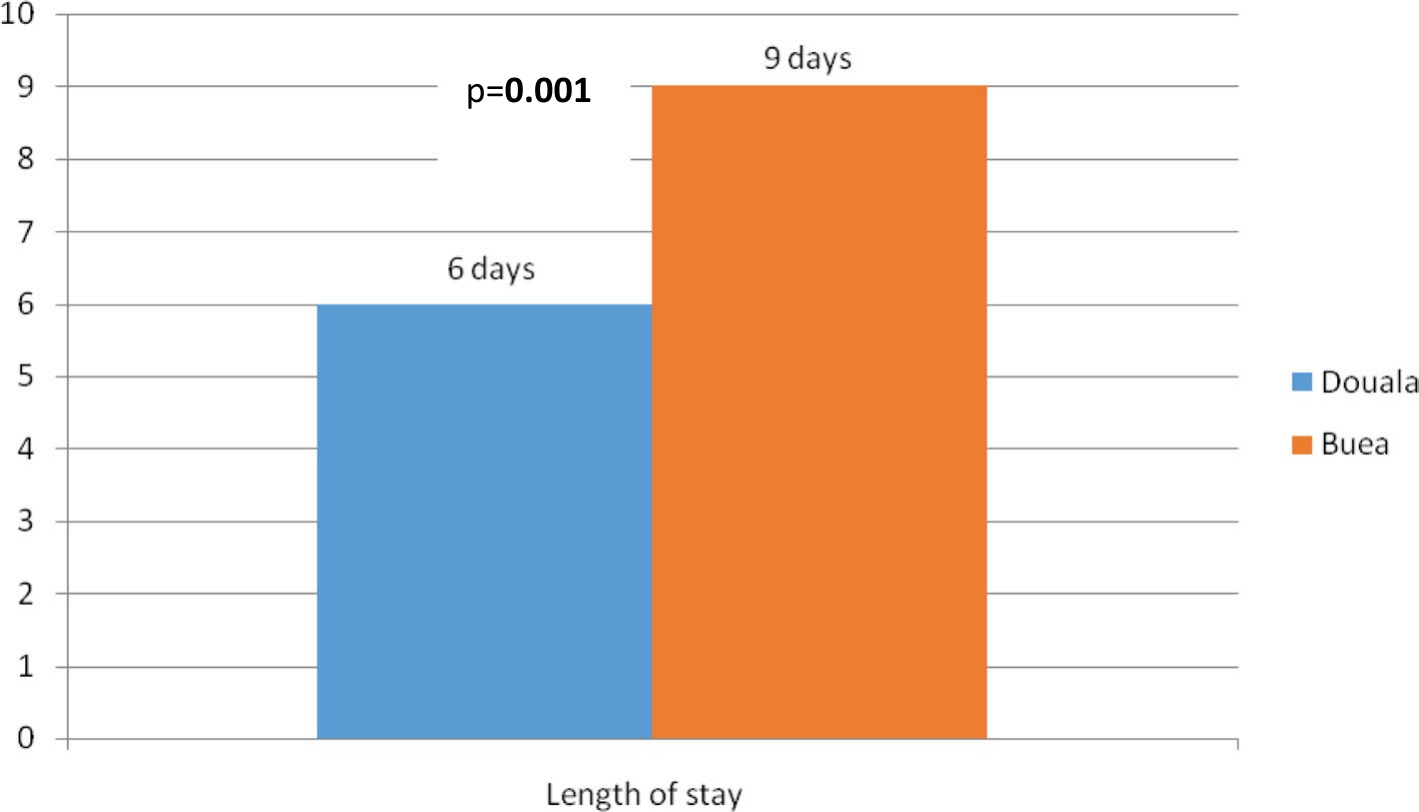
Length of stay per hospital

The median length of hospital stay **(**Fig. 3) was significantly longer in BRH 9 days (range: 0 to 49 days) than in DGH 6 days (range: 0 to 36 days), **(***p* = **0.001).** Concerning other outcomes (Table 4), one patient out of two had indications for dialysis. The need for dialysis and the access to dialysis were significantly lower in Douala compare to Buea (41.47% VS 59.09% *p* = **0.034,** 23.33% VS 73.07% *p* = **0.001).** Two patients out of three had at least a partial recovery at discharge. The mortality rate was 28%, and was relatively higher in DGH (33.18% VS 19.70%, *p* = **0.007**).

**Table 4:** Outcome of AKI of studied population

Variable	TOTAL (%) ***N*** = 349	DGH (%) ***n*** = 217	BRH (%) ***n*** = 132	p
**Need for dialysis**	168 (48.13)	90 (41.47)	78 (59.09)	**0.034**
**Access to dialysis**	78 (46.42)	21 (23.33)	57 (73.07)	**0.001**
**Kidney recovery**				
Complete	84(24.07)	61(28.11)	23(17.42)	0.066
Partial	149 (42.69)	80 (36.86)	69(52.27)	**0.006**
None	90(25.78)	56 (25.81)	34(25.76)	0.066
Unknown	26 (7.44)	20 (9.21)	6(4.55)	0.240
**Death**	98 (28.08)	72 (33.18)	26 (19.70)	**0.007**

## Discussion

To the best of our knowledge, the present study is among the first in our context to compare the causes and the outcomes of AKI in two hospitals at different level of health care in Sub Saharan Africa in Cameroon. Our data showed that AKI remains a common clinical condition in adults in urban or semi-urban areas. Patient presented with the severe form of AKI but more in the semi-urban area. Pre renal AKI was more prevalent and the main aetiologies were sepsis and volume depletion. One patient out of two needed dialysis and need of dialysis was higher in Buea. The mortality rate was higher in the DGH.

Of the 349 patients files assessed in both hospitals, 55.58% were male, resulting in an M: F ratio of1.25:1. The median age (IQR) was 54 (17–93) years, this distribution is quite similar to previous studies in other developing countries showing a male gender preponderance with those in mid-age being more affected [14–17]. This male predominance may be reflective of healthcare access bias seen in our settings. Males more frequently access hospitals due to the fact that they are usually the breadwinners and women were shown to be less likely than men to secure community-sourced healthcare financial aid [18]. In contrast, the M: F ratio in developed countries is usually 1:1 and the patients are older [19–21]. The disparities in age between developing and developed countries lies in the difference in life span of the general population (generally more longer in developed countries) and in the differences of risk factors of AKI (infectious causes versus non communicable diseases).

In this study, AKI was mainly community acquired in both centres as in literature and other studies in SSA [20, 21]. AKI in developing countries is said to be mainly community acquired and hospital acquired in developed countries, this is so because AKI is developed countries is encountered in 45% of patients admitted to the ICU and 20% of hospitalised patients, reflecting an aging population burdened by multiple comorbidities, which are often manage with multiples drugs, hypotension during anaesthesia and surgery [22] while in LMIC there is a high prevalence of communicable disease due to poverty, poor sanitation and lack of awareness [16, 23]. BRH had a significantly higher number of patients with community-acquired AKI than in DGH**.** This may be a reflection of the difference in socioeconomic level between Douala (a cosmopolitan city of about 3 million inhabitants and the most populous city in central Africa. The majority of the population lives on economic activities with a good proportion having a health insurance. The Douala town also host three hospital of first category offering up to date care) and Buea (a semi urban town where the main activity is farming). Patients mainly presented with Stage III AKI in both health institutions. Both hospitals were reference hospital and most patients presented late in hospital. Many reasons explain the late presentation: delay presentation of the patient to the primary health care, delay diagnostic and delay referral related to the absence of adequately trained health personnel to diagnose the situation or lack of finances, and sometimes even after referral the patient may take longer time to reach the appropriate hospital sometimes because of lack of finances or long distance to the hospital [16].

Sepsis, volume depletion, nephrotoxic drugs and malaria were the main aetiologies encountered in our studies. Several studies in SSA have reported similar findings [24, 25]. Sepsis and volume depletion were more often associated to AKI in DGH while severe malaria in BRH. Once more this could just be reflective of the fact that the hospitals are at different level of the pyramid of health care. Tertiary health centres rarely admit patients with malaria, has a reanimation unit so admit more severe cases like sepsis and volume depletion. Sepsis is known as an important cause of acute undifferentiated febrile illnesses in SSA and low income countries and should be part of the differential diagnosis of acute febrile illness with AKI [17]. AKI is thought to be a consequence of multi-organ dysfunction secondary to sepsis [26]. Malaria related AKI (MAKI) has been reported to be one of the leading aetiologies of AKI in SSA [20] but mostly in paediatric patients; in this study majority of patients with MAKI were in BRH reflecting here the activity of the hospital (second level of care) which expose Buea to receive more case of malaria than Douala. Nephrotoxic induced AKI was present in lower proportions (8%) with no stastical differences between both hospitals and herbal medicine was the most prevalent. Studies in SSA [27–29] and India [14] have shown higher prevalence of nephrotoxic induced AKI. In india the discribed a high prevalence of contrast, biological toxins (snake bite) and some chemicals absent in this study. Also in our setting patients rarely confess taking herbal medicine.

Majority of patients in both hospitals had prerenal AKI and acute tubular necrosis as reported by previous studies [2, 30, 31]. This reside in fact that sepsis and volume depletion being the main aetiologies will lead to pre-renal AKI and in the absence of recognition and proper management, pre-renal AKI would progress to ischemic ATN. This could also explain why DGH with the highest prevalence of sepsis recorded higher number of prerenal AKI.

The median length of hospital stay was higher in BRH (9 days) than in DGH (6 days) (*p* < 0.001). Increased level of creatinine, need for dialysis and death were shown to influence the hospital length of stay of patients with AKI [32]. BRH registered more severe kidney failure and more patients needing dialysis thus increasing the hospital lenght of stay. In the contrast, DGH registered more death thus reducing the length of stay. Access to dialysis was 46.42%, this is much lower compared to most studies in SSA (70% )[3, 20]. This lower value can be explained by the fact that many died before initiation of dialysis and the hemodynamic instabilty attributed to sepsis and hypotension may have influenced the decision of starting dialysis. This will also explain why the access to dialysis was significantly higher in Buea compare to Douala. Despite advances in medical techniques, AKI is still linked to adverse outcomes such as high in-hospital and long-term mortality rate [3]. The overall proportion of patients who died was 28.08% (*n* = 98), higher than the World AKI-associated all-cause mortality of 23.9% [23]. This difference can be explained by the presence comorbidities, the severities of underlying disease, the delay for dialysis session, the late presentation to hospital and the lack of funds. The death rate was higher in Douala than Buea (33.17% VS 19.69%, *p* = 0.007), this could be explained by the fact that DGH being a first category hospital, receved more severe cases and more sepsis related AKI as the mortality is known to be high in AKI with specific diseases when asssociated with multiorgan faillure [33, 34].

### Study limitations

As a retrospective study, quality of data was dependent on the accuracy of health provider to keep his archives. Over 30% of files were not reviewed because of either missing or incomplete clinical information. The definition of renal recovery limited to short term (at discharged), the absence of baseline serum creatinine availability may have Bias in the analysis of the outcome related to these parameters.

## Conclusion

Male,young and middle aged adult were more affected. Community acquired acute kidney injury was more present in BRH, sepsis and volume depletion, were the most frequent aetiologies in both, higher number of patients in DGH.

## Data Availability

The materials described in the manuscript, including all relevant raw data, will be freely available to any scientist wishing to use them for non-commercial purposes. The data that support the findings of this study are then available from the corresponding author (d.teuwafeu@yahoo.com) upon reasonable request.
